# Motivated Forgetting in Early Mathematics: A Proof-of-Concept Study

**DOI:** 10.3389/fpsyg.2017.02087

**Published:** 2017-12-04

**Authors:** Gerardo Ramirez

**Affiliations:** ^1^Department of Psychology, University of California, Los Angeles, Los Angeles, CA, United States; ^2^Graduate School of Education and Information Studies, University of California, Los Angeles, Los Angeles, CA, United States

**Keywords:** motivated forgetting, affect, memory retention, identity threat, math anxiety

## Abstract

Educators assume that students are motivated to retain what they are taught. Yet, students commonly report that they forget most of what they learn, especially in mathematics. In the current study I ask whether students may be motivated to forget mathematics because of academic experiences threaten the self-perceptions they are committed to maintaining. Using a large dataset of 1st and 2nd grade children (*N* = 812), I hypothesize that math anxiety creates negative experiences in the classroom that threaten children’s positive math self-perceptions, which in turn spurs a motivation to forget mathematics. I argue that this motivation to forget is activated during the winter break, which in turn reduces the extent to which children grow in achievement across the school year. Children were assessed for math self-perceptions, math anxiety and math achievement in the fall before going into winter break. During the spring, children’s math achievement was measured once again. A math achievement growth score was devised from a regression model of fall math achievement predicting spring achievement. Results show that children with higher math self-perceptions showed reduced growth in math achievement across the school year as a function of math anxiety. Children with lower math interest self-perceptions did not show this relationship. Results serve as a proof-of-concept for a scientific account of motivated forgetting within the context of education.

## Introduction

Despite all of the effort that students put into studying, they commonly report that knowledge is rapidly lost once a course is over. While the belief in a total loss of formally acquired knowledge is false (Bahrick, 1979), it is true that students experience a significant amount of forgetting soon after the completion of a course (Conway et al., 1991; Kamuche and Ledman, 2011). Research on long-term retention of classroom knowledge reports that forgetting arises because of blocked learning schedules (Landauer and Bjork, 1978; Dempster, 1992), a lack of subsequent relearning (Bahrick and Phelps, 1987; Bahrick and Hall, 1991; Cooper et al., 2000; Deslauriers and Wieman, 2011), poor initial knowledge structures and shallow levels of understanding gained during the course itself (Conway et al., 1991). In this article, I consider an alternative explanation. I draw on the suppression and threat based coping literature to argue that students themselves may be motivated to forget due to negative academic experiences that threaten their self-perceptions.

### Motivated Forgetting

Motivated forgetting is the active process of forgetting memories that are unpleasant, painful, or generally threating to the self-image that individuals strive to maintain (Tajfel and Turner, 1986; Thompson et al., 1997). Research in cognitive psychology demonstrates that people can intentionally down-prioritize unwanted memories from entering consciousness via control processes. For instance, studies using the think-no-think paradigm (Anderson and Green, 2001; Anderson and Levy, 2009) demonstrate that individuals are capable of intentionally forgetting memories for words that were previously encoded. The think-no-think paradigm begins by requiring students to first learn a list of cue-target pairs (e.g., Ordeal-Roach). After this initial learning phase, individuals are presented with a previously studied cue (i.e., Ordeal) and are instructed to either remember the associated target word (i.e., Think of Roach) or to suppress the associated target word (i.e., No-think; Roach). During a memory test at the end, individuals are again presented with the same cues and asked to try their best to recall the correct target memory words. These studies consistently reveal that individuals have more difficulty recalling words that they were previously instructed to suppress relative to words they were instructed to remember. Additionally, individuals are also worse at recalling suppressed words relative to baseline words which were previously studied but were not paired with either “remember” or “suppress” instructions.

Neuroimaging studies focused on intentional forgetting (Anderson et al., 2004; Benoit et al., 2015) reveal that suppressing memory items during no-think trials is associated with higher activity in brain regions involved in inhibition (e.g., lateral prefrontal cortex and anterior cingulate cortex) as well as reduced activity in regions involved in episodic memory (e.g., the hippocampus). Research using the think-no-think paradigm, as well as related research using directed forgetting (Bjork and Bjork, 2003) and retrieval-induced forgetting paradigm (Storm and Jobe, 2012) ultimately reach a similar conclusion: individuals are capable of intentionally forgetting information that has been entered into memory (Bjork et al., 1968, 1998; Davis and Okada, 1971; MacLeod, 1975; Depue et al., 2006).

### Threat Based Model of Motivated Forgetting

The cognitive literature on intentional forgetting provides compelling evidence that forgetting previously encoded information is possible, yet it has not provided an account for why individuals engage in intentional forgetting outside of the laboratory. Recent social psychological investigations have advanced the motivated forgetting literature by developing a framework to predict the social and intrapersonal circumstances that elicit motivated forgetting. A growing body of research draws on threat-based theories to argue that motivated forgetting arises as a possible coping mechanism to defend against memories that threaten the integrity of the self (Tajfel and Turner, 1986; Aronson et al., 1999; Sherman and Cohen, 2006). Threat-based theories begin with the premise that people are motivated to see themselves in an overly positive light and will fundamentally alter their behaviors, attention, and even memory processes to maintain this positive internal representation (Greenwald, 1980; Taylor and Brown, 1994).

Threat-based theories argue that when individuals experience a threat to their mental representation of the self, they deploy self-enhancement coping strategies designed to reduce the source of the threat. For instance, individuals whose positive domain identity is under threat will fail to encode threatening information (Appel et al., 2011), disidentify with a domain (Osborne, 1997; Major et al., 1998; Schmader and Major, 1999; Osborne and Walker, 2006; Nussbaum and Steele, 2007) or reduce their interest in a threatened domain (Cheryan and Plaut, 2010). Engaging in motivated forgetting is another way individuals cope with threatening situations (Sedikides and Green, 2004; Sahdra and Ross, 2007; Imhoff and Banse, 2009; Rotella and Richeson, 2013). For instance, individuals show selective forgetting of behavioral feedback when that feedback is negative rather than positive in valance and deals with central rather than peripheral aspects of the self (Green and Sedikides, 2004). By contrast, individuals are equally good at recalling negative and positive behavioral feedback about others (Sedikides and Green, 2004; Newman et al., 2016). There is also evidence indicating that students who cheat on a laboratory task clear their conscience by forgetting an honor code designed to promote academic honesty (Shu et al., 2011; Shu and Gino, 2012) as well as the details of the context where the cheating took place (Kouchaki and Gino, 2016).

Motivated forgetting is a powerful defensive reaction that seeks to reduce conscious awareness of various aspects of experiences that threaten the self. Moreover, individuals with a particularly favorable view of the self for a given domain seem to be even more susceptible to reacting defensively against the threats that challenge their personal perceptions (Baumeister et al., 1996; Jones et al., 2002). For instance, students forget even relatively neutral information that has been linked to their school after being presented with information that questions the academic status of their school (Dalton and Huang, 2014). However, these effects only occur for students who begin the experiment with a strong school identity for whom information that casts doubt on the academic standing of their universe would be received as a threat. Similarly, individuals remember events happening in the distant past when these events are negative and they are motivated to maintain favorable self-view (Ross and Wilson, 2002). The social psychology literature on motivated forgetting suggests is that interpersonal differences and specific social contexts can be helpful in predicting which subgroups of students are susceptible to engaging in defensive coping mechanisms, like motivated forgetting, when under threat.

### Motivated Forgetting and Academic Break Periods

A recent study examined the consequences of motivated forgetting within the context of education, where students are generally motivated to remember. Ramirez et al. (2017) asked whether we can draw on the basic premises underlying the research on psychological threat and motivated forgetting to predict the circumstances under which students forget course content once the class is over. To test this, students enrolled in a multivariable calculus course were asked to report their math self-perceptions and course relevant stress. At the end of the quarter, students completed their final exam per usual which served as a baseline measure of acquired knowledge. The authors measured how much content students forgot by asking them to re-take some items from their final exam 2 weeks into the summer break.

Ramirez et al. (2017) theorized that higher course-related stress would predict greater forgetting of course content during the summer, and that this relationship should only be evident among students with high math self-perceptions. This is exactly what was found. It was suggested that students who believe themselves to be quite competent in math appraise the high rate of ongoing class stress as a threat to their self-perception (e.g., “Mathematics is supposed to be something I am good at, why am I feeling so stressed out by this class?”). This threatened state is thought to create a defensive coping mechanism that leads to a motivation to forget once the class is over (i.e., during the summer break period). Interestingly, they also found that students who claimed to have a more stressful classroom experience reported that they actively avoided consciously thinking about their course during the summer. But once again, the relationship between course stress and avoidance of course-related thoughts was only found for students higher in math self-perceptions. This pattern of results fits well with contemporary suppression and mental context shift models of motivated forgetting (Sahakyan and Kelley, 2002; Dalton and Huang, 2014; Benoit et al., 2015; Manning et al., 2016).

As stated earlier, students under threat can engage in a host of other defensive responses meant to reestablish their self-image (Steele et al., 2002; Necka et al., 2015). For instance, students might deal with threat by disengaging from the class or processing the material poorly from the start (Appel et al., 2011). If this is the case, then it seems possible that the aforementioned motivated forgetting effects could be due to more general difficulties with learning the material which should be evident in students’ initial final exam performance (what might be termed a “deficient encoding hypothesis”). The results of the previous investigation did not support this account (Ramirez et al., 2017). Students who were at risk for experiencing motivated forgetting did not perform differently on the initial final exam from those students who were not at risk for motivated forgetting. But once the course was over, students at risk for motivated forgetting both quickly forgot what they learned in class and consciously avoided thinking about their course experience.

Ramirez et al. (2017) focused on the summer period following the completion of a course because they argued that students would only instantiate their motivation to forget once they felt that there were no longer immediate consequences for doing so (i.e., at the completion of a course and start of a long summer break). Students commonly engage in forgetting only when they interpret cues from various educational practices that the previous material they encountered is no longer relevant (Rohrer and Taylor, 2007; Bunce et al., 2011; Lawrence, 2013). Academic rest periods can be one such cue; they serve as sharp event boundaries (Radvansky and Copeland, 2006; Radvansky et al., 2011) that encourage students to transition into maintaining rest and leisure as opposed to work and effort goals. In fact, prior work shows after working on a difficult activity, individuals show heightened accessibility of rest-related semantic concepts and leisure activities (Job et al., 2015).

A heightened focus on maintaining leisure vs. work goals may down-regulate personally relevant threatening memories. For instance, accruing neuro-scientific evidence shows that rest and other non-attentive mental states lead people to become absorbed in free-form thought that is associated with internal self-reflection, maintenance of self-knowledge, autobiographical planning, and retrieval of personal memories (Gusnard et al., 2001; Ochsner et al., 2005; Amodio and Frith, 2006; Stawarczyk et al., 2011; Immordino-Yang et al., 2012). Students also show enhanced activation in resting state neural regions after receiving a threat to their identity which in turn leads to better coping and moderates the extent to which threats influence performance perceptions (Forbes et al., 2015).

Taken together, these findings lead to a novel conception of the self-relevant memory processes that individuals engage in when they are “at rest.” Extensive academic rest periods – which allow students to feel they can let go of previous work goals – may enhance rest-related neural maintenance activity, leading students to engage in psychological defensive adaptations of threatening educational memories.

### Current Study

To summarize, memory research suggests that people are capable of intentional forgetting. Threat-based theories identify self-perceptions and experiences that threaten self-perceptions as key ingredients in creating a defensive memory reaction that leads to a motivation to forget. Recent neuro investigations of threat suggests that brain activity during rest periods is associated with activation in areas involved in defensive memory adaptations. The aim of the current proof-of-concept study is to address whether children at risk for motivated forgetting may be forgetting important course relevant content during academic break periods.

I focus on examining how individual differences in students’ math anxiety (i.e., fear for situations that involve math) and math self-perceptions (i.e., their perceptions of how interesting and enjoyable it is to engage in math) interact to create threat. It is actually quite common for young students to hold high math performance and ability self-perceptions while also maintaining a high degree of math anxiety (Foley et al., 2017). Math anxiety and math self-perceptions have been shown to be related but distinct dimensions of math affect (Bai et al., 2009; Krinzinger et al., 2009). Building off previous research on early math anxiety (Devine et al., 2012; Young et al., 2012; Ramirez et al., 2013, 2016; Vukovic et al., 2013; Carey et al., 2016; Hill et al., 2016), I surmise that children high in math anxiety are likely to experience disfluent math experiences throughout the school year and that for those high in math self-perceptions, these disfluent experiences will create identity threat.

Previous research suggests that children are vulnerable to identity threat (Ambady et al., 2001; McKown and Weinstein, 2003; Galdi et al., 2014). Young children in elementary school, in particular, tend to have overly optimistic perceptions of how much they are personally interested in and competent in math (Nicholls and Miller, 1984; Eccles et al., 1993; Fredricks and Eccles, 2002). These optimistic perceptions appear to be based on wishful thinking (Stipek and Mac Iver, 1989) which could put early elementary children at risk for possessing the interpersonal ingredients (i.e., high math anxiety and high math self-perceptions) that renders one vulnerable to threat-based motivated forgetting.

A novel aspect of the current study is that I focus on motivated forgetting as it might occur in the middle of the 9 months academic term rather than at the end of the school year. Why would children forget important course-related content when they are still in the midst of the 9 months academic term? A large majority of children in the United States undergo an extensive rest period during the middle of December. Prior research finds that extensive rest periods, in both summer and winter, can lead children to lose recently gained knowledge (von Hippel, 2007). Sociologist attribute this learning loss primarily to inadequate access to resources and formal opportunities to learn math during the rest period (Heyns, 1978; Cooper et al., 2000; Douglas et al., 2004). However, I argue that the winter break may also create a sharp cue for rest and leisure goals that instantiates the motivated forgetting of students under threat from their previous class experience.

Traditionally one could operationalize forgetting as a change in children’s performance from baseline to some future time period after instruction has ended. Another approach is to infer forgetting by measuring reduced growth in math achievement across the school year under the premise that forgetting should result in diminished progress we might otherwise expect. This study employs both approaches to studying forgetting. I begin by examining reduced math achievement growth using standardized residuals produced from a model of fall math achievement predicting spring math achievement. Fall achievement is a strong predictor of spring math achievement, which makes the use of residual scores a meaningful way to quantify how children perform in the spring relative to what we would expect given their fall achievement score. This residual growth approach is a common method for measuring progress in value-added analyses within the field of education. I reason that children who show greater forgetting during the winter break period will have to work harder to make up that knowledge loss and display reduced growth in math achievement across the school year. Given that all children undergo a winter break period in the middle of the school year, I predict that children who are at risk for motivated forgetting will show standardized spring achievement scores that are below what we would expect given their fall math achievement (i.e., negative residual achievement scores).

A drawback to using residual achievement scores is that they hide whether a negative residual score occurs simply because children are growing less than the model average would predict, or whether they are actually performing worse in the spring relative to the fall assessment. To address this, I also take a more traditional difference approach to measuring forgetting by subtracting fall achievement from spring achievement where positive scores indicate growth and negative scores indicate forgetting. I use this difference scoring approach to categorize children as belonging to a group who either show growth or loss (i.e., forgetting) in achievement from fall to spring. If children under threat are truly forgetting, then I predict that they should show greater negative residual achievement scores and a higher probability of belonging to a group of children who show losses in their math achievement from fall to spring.

Lastly, I also evaluate whether the motivated forgetting account proposed might be better explained by a deficient encoding hypothesis in which children under threat are simply learning the material less during school year. To test this alternative account, I leverage the delay between the start of the school year and the first fall assessment session as a measure of students’ learning prior to entering the winter break. If children who are at risk for motivated forgetting are simply learning less well in general, then we should expect those higher in math self-perceptions to show reduced achievement in the fall as a function of math anxiety.

## Materials and Methods

### Dataset and Participants

Data for this proof-of-concept study came from a larger project examining how student learning relates to classroom practices (Maloney et al., 2015). It is important to note that the larger project was not originally designed to test a motivated forgetting account of mathematics. To conduct analyses, I drew upon existing measures within the larger project to test my hypotheses. Data were collected from a diverse sample of children attending elementary schools in a large urban setting. This study was carried out in accordance with the recommendations of Social and Behavioral Sciences Institutional Review Board at University of Chicago. Parents of child participants signed an informed consent form. Parents who consented for their child to be involved also completed a short questionnaire that included demographic items. Our final sample of children consisted of *N* = 812 students (*n* = 365 boys; *n* = 356 first grade) who had completed all of the measures for both the fall and spring sessions and did not show problems during data collection. The ethnic breakdown of students was as follows: 31.8% were African American, 23.4% White, 24.9% Hispanic or Latino, 6.4% Asian and 6.4%were other/mixed race. Ethnic information for 6.9% of students was not reported by parents.

### Measures

Trained research assistants conducted the assessment sessions with children in the fall season and spring season. The fall session took place within the first 3 months of the school year, while the spring session took place within the last 2 months of the school year. For both fall and spring, math achievement was assessed as a part of a broader achievement assessment focused on children’s cognitive skills and competencies. Children’s math self-perceptions and math anxiety were assessed as a part of an affect session focused on children’s math attitudes around schooling. The achievement session always occurred first. The same set of achievement and affect measures were also taken in the spring. All of the sessions were completed on school grounds.

*Math Achievement* was measured using a normed standardized test of math skills which has been found to shown good construct validity and relate to established practice in schools (The Woodcock–Johnson III; Woodcock et al., 2001). Math achievement was specifically measured using the Applied Problems subtest which presents children with math related word problems (e.g., if you have seven crayons and someone takes away 3, how many do you have left?) that increase in difficulty as children progress. Research assistants began by establishing basal (six items in a row correct) and ended the session once children reached ceiling (six items in a row incorrect within the same page). The Applied Problems Form A was used in the fall and a parallel form containing different problems (Form B) was used in the spring. The applied problem subtest was introduced to children as a number game. The Applied Problems subtest shows internal-consistency reliability above 0.80 between first and third grades (Jordan et al., 2010). For both fall and spring math achievement, I used the *W*-scores which are transformations of the raw score into a Rasch-scaled score with equal intervals.

*Math anxiety* was measured using a revised child math anxiety questionnaire (CMAQ-R). The CMAQ-R was developed to be appropriate for children in 1st and 2nd grade (Maloney et al., 2015; Ramirez et al., 2016) and involves presenting children with 16 items that ask how nervous they feel toward various situations that require math-related interactions. The wording of the scale is prospective (e.g., How do you feel when you have to sit down and start your math homework?). Children responded to the scale by pointing to one of five cartoon faces that represent not nervous at all (1) to very, very nervous (5). The CMAQ-R shows good reliability (*alpha* = 0.83).

Math self-perceptions were measured using three items from the Trends in International Mathematics and Science Study (TIMSS; PISA, 2013). The items are: (1) I enjoy learning mathematics, (2) I like mathematics, and (3) Mathematics is boring (reversed scored). These three items are interpreted to relate to children’s subjective task value within Eccles’ expectancy-value theory (Wigfield and Eccles, 2000). For ease of interpretation, I refer to these items as “math self-perceptions.” The three items showed acceptable reliability (*alpha* = 0.75) for a young sample of children. The math self-perception items as well as the CMAQ-R items were introduced to children as a question game.

## Results

I began by regressing spring Math *W*-scores onto fall math *W*-scores in order to generate standardized math achievement residual scores, which served as my primary outcome variable. As we might expect, fall math *W*-scores were a significant predictor of spring math *W*-scores (*r* = 0.811, *t* = 39.49, *p* < 0.01, *adjusted r-square* = 0.65). The math residual scores that were generated estimate the extent to which fall math *W*-scores predict spring math *W*-scores in standardized residual units. Math residual scores above zero indicate that the actual spring *W-*score is higher than predicted by the fall math *W*-score (i.e., a score that is above the prediction line). Math residual scores below zero indicate that actual spring *W*-scores are lower than predicted by the fall math *W*-score.

**Table 1** shows the correlations of the variables of interest. I note a couple of relationships. First, children’s math self-perceptions were a weak predictor of children’s fall math achievement and their growth in math achievement. Children’s math anxiety was a significant predictor of fall math achievement, which provides preliminary evidence of the premise that math anxiety may contribute to disfluent learning experiences that create a feeling of threat.

**Table 1:** Correlation coefficients for main variables of interest.

Variable name	Mean	*SD*	1	2	3	4	5
(1) Fall math *W*-score	463.76	21.43	–				
(2) Math anxiety	2.47	0.78	-0.26^∗∗^	–			
(3) Math self-perception	3.43	0.76	0.08^∗^	-0.19^∗∗^	–		
(4) Residual math score	0.01	1.00	0.01	-0.09^∗∗^	-0.03	–	
(5) Grade	1.56	0.50	-0.42^∗∗^	-0.16^∗∗^	-0.01	-0.06	–
(6) Gender	1.45	0.50	-0.03	-0.05	0.03	-0.01	-0.02


*^∗^*p* < 0.05, ^∗∗^*p* < 0.01, ^∗∗∗^*p* < 0.001.*

The two main predictor variables were mean centered before multiplying them together to create the interaction term. I then checked for some basic model assumptions. Multicollinearity did not appear to be an issue with the data given the weak inverse correlation between math anxiety and math self-perceptions (*r* = -0.19, *p* < 0.01) and VIF values between 1 and 2. I also plotted the model residuals in a histogram and found visual evidence for a normal distribution of residuals. Plotting the standardized predicted values against the standardized residuals revealed that there were no systematic patterns of clustering to warrant concern for homoscedasticity. The Durbin–Watson test value (*d* = 1.797) indicates that there is no evidence of autocorrelation. The between-classroom variance estimates for the residual math score was not significant (*p* < 0.01); thus analyzing the data hierarchically was not necessary. Lastly, I found that missing data (<6% of the data) was missing completely at random using Little’s Missing Completely at Random test (Chi-Square = 21.646, DF = 19, *p* = 0.302).

The main research question was addressed using a series of simultaneous regression models using PROCESS (Hayes, 2013). PROCESS uses a listwise deletion procedure to deal with missing data. I began by evaluating whether children’s math anxiety (the predictor variable) relates to math residuals score (the outcome variable) and whether this relation is moderated by math self-perceptions (the moderator variable). I found a significant effect of math anxiety (*b* = -0.14, *t* = -3.13, *p* = 0.002) but not math self-perceptions (*p* = 0.39). Critically, I also found that the math anxiety × math self-perceptions term was a significant predictor of residual scores for math (*b* = 0.16, *t* = -2.95, *p* = 0.003). The results are presented in **Table 2** (model 1).

**Table 2:** Regression models predicting the math residual score and the fall math achievement.

	Math residual scores			Math residual scores			Fall math achievement		
			
	(Model 1)			(Model 2)			(Model 3)		
			
Variable	*B*	*SE B*	*β*	*B*	*SE B*	*β*	*B*	*SE B*	*β*
Constant	-0.01	0.04		-0.13	0.16		463.66	0.74	
Math self-perceptions (MSP)	-0.04	0.05	-0.03	-0.04	0.07	-0.03	1.04	0.99	0.04
Math anxiety (MA)	-0.14	0.05	-0.11^∗∗^	-0.14	0.07	-0.11^∗∗^	-6.92	0.95	-0.25^∗∗^
Interaction: MA × MSP	-0.16	0.05	-0.15^∗∗^	-0.15	0.05	-0.10^∗∗^	-1.17	1.1	-0.04
Gender				0.01	0.05	0			
Grade				0.07	0.05	0.03			
*R-square*		0.02			0.02			0.06	
*F*		5.95			3.75			19.4	


*^∗^*p* < 0.05, ^∗∗^*p* < 0.01.*

**FIGURE 1:**
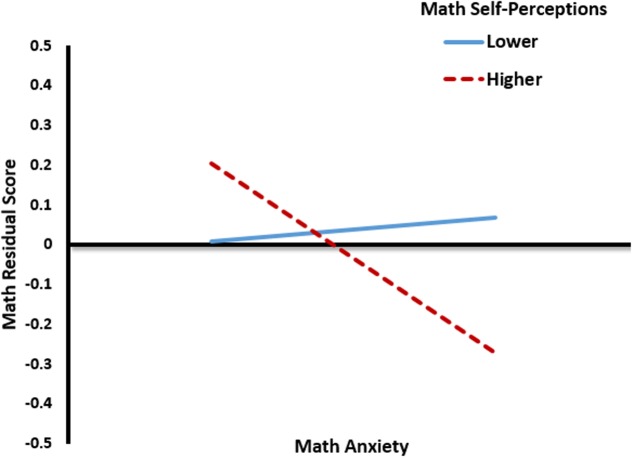
Interaction between math anxiety and math self-perception on math residual scores. Math anxiety and math self-perceptions are plotted at the 10th and 90th percentile.

To explore the interaction, I examined the effects of math anxiety on residual scores for math at the 10th and 90th percentile of math self-perceptions (see **Figure 1**). A simple slopes analysis revealed that children higher in math self-perceptions demonstrate an inverse relationship between math anxiety and residual scores for math (*b* = -0.231, *t* = -4.06, *p* < 0.01). That is, children with higher on math anxiety and math self-perceptions had a spring math *W*-score that was lower than what we should expect given fall math achievement. By contrast, children with high math self-perceptions and low anxiety showed spring achievement that was higher than expected, given their fall math achievement. For children lower in math self-perceptions, the relationship between math anxiety math residual scores was not significant (*p* = 0.68).

To ensure that the effects in model 1 were not being driven by either dispersion of grade or gender, I next re-ran model 1 while holding grade and gender constant. The effects in model 1 hold even after simultaneously controlling for children’s gender and grade (see model 2 in **Table 2**). I removed gender and grade from all subsequent analyses as they were not significant covariates.

Another way of evaluating whether the achievement growth results reported above might be driven by forgetting *per se* involves examining whether children showed growth vs. loss from fall to spring. It is actually quite common for longitudinal achievement datasets to report that some students show a reduction in achievement during post assessments. The current dataset had subsample of *n* = 158 students who showed a reduction in achievement *W*-scores from fall to spring. This interesting pattern of achievement loss allowed me to ask whether children at risk for motivated forgetting might show a probability of being represented by this subsample. I began by subtracting Spring math *W*-scores from Fall Math *W*-score to create a loss score which coded children who showed either some to no growth as (0) against children whose spring score was lower than their fall score as (1). A logistic regression was run with math self-perceptions, math anxiety and the interaction term predicting probability of belonging to the group of children who showed a loss in achievement from fall to spring. A test of the full models against a constant only model was statistically significant (χ^2^ = 16.19, *p* < 0.01, *df* = 3). Results reveal a significant effect of fall math anxiety (*wald* = -2.63, *p* < 0.01), math self-perceptions (*wald* = -2.61, *p* < 0.01) as well as the two way interaction (*wald* = 3.13, *p* < 0.01). A simple slopes analysis among children high in math self-perceptions (i.e., at the 90th percentile) revealed a significant positive relationship between math anxiety and the probability of belonging to the sub-group of children who demonstrate a loss in achievement from fall to spring (*wald* = 3.43, *p* < 0.01). Children lower in math self-perceptions (i.e., at the 10th percentile) did not show a similar association (*p* = 0.13)^1^.

One explanation for the pattern of results reported in model 1 is that children at risk for motivated forgetting are learning the material less well to begin with, which leads their fall achievement measure to serve as a poor predictor of spring math achievement. One way of evaluating this involves examining children’s math achievement in the fall when learning took place prior to the winter break. To do this, I first generated a fall gap score, which quantifies the amount of days between when children started the school year in their respective school and when they were tested in the fall. Children’s fall assessment occurred, on average, about 2 months after the start of school suggesting that enough time has elapsed to allow for individual differences in learning (*M* = 57.91, *SD* = 27.70). Hence, I regressed fall math *W*-scores on the same set of factors outlined in model 1. Results reveal that only the math anxiety coefficient was a significant predictor of math *W*-scores (*b* = -6.92, *t* = -7.28, *p* < 0.01). Math self-perceptions as well as the interaction between math anxiety and math self-perceptions were not significant (*p* = 0.29 for both), which suggests that students at risk for motivated forgetting were not learning less to begin with (see model 3 in **Table 2**). The correlational results in **Table 1** also reveal that there wasn’t a relationship between fall math achievements and the math residual score, suggesting that children with lower achievement in general did not show less growth across the year.

## Discussion

What students learn in school is assumed to accumulate and provide a foundation for future coursework. However, anecdotal reports as well as empirical investigations suggest that students forget much of what they are taught soon after a course concludes (Bahrick, 1979; Bahrick and Hall, 1991). In fact, extensive break periods during the summer and winter have been shown to contribute to a profound forgetting rate (Cooper et al., 1996; von Hippel, 2007). The aim of this proof-of-concept study was to evaluate whether part of the forgetting rate that occurs during the winter break is due to students’ very own motivation to forget.

Results show that among children with high math self-perceptions, greater reported math anxiety was associated with a spring achievement score beneath what would be expected given the fall math achievement score (i.e., a lower residual achievement score). Interestingly, children with lower math self-perceptions did not show such a relationship. A similar pattern of results was also found when analyzing whether children belonged to a group who show a loss in achievement from fall to spring. I contend that a high degree of math anxiety threatens children’s high math self-perceptions and that this threat leads to a motivation to forget, which contributes to reduced growth in achievement.

The motivated forgetting account put forth is grounded in a large body of basic laboratory research demonstrating that intentional forgetting is not only possible (Anderson et al., 1994) but can also occur as an adaptive process to reduce the accessibility of irrelevant information that no longer provides utility or value (James, 1890; Bjork, 1989). Social psychological research highlights the interpersonal differences and contexts that lead some students to be willing and active participants in this forgetting process. For instance, threat-based theories contend that individuals with a highly favorable view of the self for a given domain engage in a host of adaptive responses meant to reduce threats to the self (Sherman and Cohen, 2006). Recent investigations suggest that one way students respond to threats is by engaging in motivated forgetting.

My focus on examining forgetting via a reduced growth in achievement during the 9 months academic term raises an important question: Why would children forget academically relevant content that is still relevant for current and future courses? I argue that the winter break period can lead children to the erroneous conclusion that it is okay to forget the material they previously encountered since it holds no immediate relevance (Bjork et al., 1998; Bunce et al., 2011; Lawrence, 2013). Research on event cognition (Radvansky and Copeland, 2006; Radvansky et al., 2011) as well as context shift forgetting (Sahakyan and Kelley, 2002; Jonker et al., 2013) suggests that as students transition from the fall term into the winter break, a sharp context transition may take place which makes students prone to forget more information than if they had never made that shift.

A shift from a work context (during the fall academic term) into rest and leisure contexts (during the winter break) also might lead students to maintain a different set of goals. For instance, previous research reveals that individuals who have exerted themselves show heightened activation of semantic rest concepts as well as preference for rest-conducive objects and actual resting behavior (Job et al., 2015), suggesting that an active motivational and attentional process are involved in maintaining rest and leisure relevant goals. Rest-relevant goals during extensive break periods may act to down-prioritize both work related concepts as well as reduce the accessibility of unwanted work related memories from entering consciousness, thereby helping to carry out an individual’s motivation to forget. In line with this account, it has been previously found that students at risk for experiencing motivated forgetting report that they actively avoid thinking about the course material during the summer break period (Ramirez et al., 2017). Moments of inactivity appear to be periods when individuals attempt to cope with self-oriented threats (Lyons and Beilock, 2012; Forbes et al., 2015).

The motivated forgetting interpretation presented is, of course, open to several alternative accounts, which I attempt to address. One alternative account is that children high in math self-perceptions started the fall with high math achievement and hence had less to gain across the school year. This interpretation would help explain why children with high math self-perceptions showed reduced growth as a function of math anxiety. However, it is curious to note that math self-perceptions was only weakly related to fall attachment and did not relate to math residual scores. The math self-perceptions measure seem to be an indicator of children’s affective disposition toward mathematics rather than a predictor of their actual ability in the domain. Children’s math self-perceptions were also only weakly related to math anxiety, which provides evidence that they are distinct constructs that interact in a meaningful way to create threat-based motivated forgetting.

Another alternative account is that students under threat may have learned the material less well before they went on winter break, which put them at risk for reduced achievement growth throughout the school year (i.e., deficient encoding hypothesis). For instance, laboratory studies show that individuals cope with stress and threat by directing attention away from threatening stimuli (Papies et al., 2008) as well as by taking less effective academic notes (Appel et al., 2011) relative to their non-threatened peers. Research on expectancy × value interaction (Trautwein et al., 2012) would also predict that individuals with low expectation for success (i.e., math anxious children) should show a negative relationship with achievement that is more pronounced among those with high subjective task value (i.e., children with high math self-perceptions). A strong test for both of these alternative accounts is to examine the math self-perceptions × math anxiety interaction for children’s fall math achievement specifically. After all, the fall achievement session took place almost 2 months after the start of school, which would presumably provide enough time for fall math achievement scores to serve as an index of how well children learned math before going into winter break. The results revealed that children’s fall math *W*-scores were well predicted by math anxiety in general but this relationship did not vary as a function of individual differences in math self-perceptions. Hence, it does not appear to be the case that children under threat learned the material in a deficient manner or were less motivated. Rather, I argue that children engaged in threat-based motivated forgetting which affected students’ growth in math achievement.

### Limitations

The current study holds several limitations which warrant attention. For instance, the data collection timeline makes it difficult to make a strong case that the winter break period was responsible for allowing students to engage in motivated forgetting; all of the students in the study underwent a winter break period. Future research should examine achievement immediately prior to and after the winter break period using assessments that measure what children are learning in their specific class. The reliability for my measure of math self-perceptions could have been higher if children had been presented with more items, as well. The correlational research design also did not allow me to manipulate extensive rest periods or identity threat, which could have provided causal evidence for the account I put forth. Lastly, I was also not able to assess perceived threat, intention to forget, or suppression-avoidance processes, which limited my ability to provide greater evidence for the mechanism of motivated forgetting.

As it is, there already exist experiments that systematically manipulate threat within a carefully controlled laboratory environment (Sedikides and Green, 2004; Shu et al., 2011; Shu and Gino, 2012; Dalton and Huang, 2014; Kouchaki and Gino, 2016; Newman et al., 2016). I drew upon the well-established social psychological work on motivated forgetting to evaluate the necessary ingredients thought to create threat. My goal was to provide a proof-of-concept for the notion that motivated forgetting processes may play a role in the retention of educationally relevant material. There are no studies examining what affective factors might be responsible for the retention of math knowledge. The purpose of this study is to open up a window into the defensive reactions that might be responsible for the retention of education knowledge that students are often committed to maintaining.

A promising direction for future research is to experimentally test the different conditions under which motivated forgetting might arise in an educational context. For instance, are extensive academic break periods necessary to promote students indulging in their intentions to forget or might we find similar effects among students who simply believe the material holds very little value and relevance to their lives? I discuss other future directions below.

### Implications and Future Directions

The work reported here extends research on motivated forgetting to an educational context where students typically exert a great deal of effort to avoid forgetting. This work also makes a novel contribution by providing evidence that even young children are capable of experiencing threat-based motivated forgetting. If motivated forgetting is indeed a factor reducing children’s accessibility of memories for important course content, then ensuring that children begin and leave the classroom with a restored sense of self (Walton, 2014) could be quite useful in reducing motivated forgetting.

Children are regularly engaging in sense making processes meant to reduce internal dissonance between what they believe about themselves and their ongoing experience (Egan et al., 2007). While dissonance experiences can be quite powerful toward helping children to undergo conceptual change (Guzzetti et al., 1993), we should also redirect children toward a more optimal appraisal of educational experiences that challenge their self-perceptions. Indeed, individuals who engage in story editing are better able to adjust to ongoing difficult circumstances (Wilson and Linville, 1982). Instructors may help students to discover the utility value of the material they are encountering (Hulleman and Harackiewicz, 2009), especially at the conclusion of the course. Students may also be less motivated to forget content, even content that is perceived as threatening, if they engage in self-generating insights into how the knowledge they are learning will continue to be relevant in their lives (Canning and Harackiewicz, 2015). Teachers and parents could also help children to view anxiety provoking and disfluent learning experiences as ideal for growth (Wilson and Linville, 1982; Jamieson et al., 2013; Haimovitz and Dweck, 2016), which could reduce maladaptive interpretations of failure.

## Conclusion

Taken together, the promising findings of this proof-of-concept study suggests that children may deal with threatening classroom experiences by forgetting important course relevant knowledge. Although there has been a recent interest in using a motivated forgetting framework to understand defensive adaptations in the social psychology literature (Shu et al., 2011; Dalton and Huang, 2014; Kouchaki and Gino, 2016), the work reported here and elsewhere (Ramirez et al., 2017) is the first to extend research on motivated forgetting to the classroom. These findings lead to a novel conception of the defensive adaptations that students engage in when they are “at rest” from schooling.

## Author Contributions

The author confirms being the sole contributor of this work and approved it for publication.

## Conflict of Interest Statement

The author declares that the research was conducted in the absence of any commercial or financial relationships that could be construed as a potential conflict of interest.

## Footnotes

1 A similar pattern of results were obtained when I predict student s’ spring achievement after adjusting for fall achievement. The main effect of mathematics self-concept (*b* = -0.56, *t* = -0.85, *p* = 0.40), math anxiety (*b* = -2.07, *t* = 3.15, *p* = 0.002) and the interaction term were all significant (*b* = -2.19, *t* = -2.97, *p* = 0.003; adjusted *r*-square = 0.66).
